# Supplementary material for: A Novel Pathway of Chlorimuron-Ethyl Biodegradation by Chenggangzhangella methanolivorans Strain CHL1 and Its Molecular Mechanisms
Source: Int J Mol Sci. 2022 Aug 31;23(17):9890. doi: 10.3390/ijms23179890 (PMC9456165; doi:10.3390/ijms23179890)
Supplement: Supplementary file 1 [file ijms-23-09890-s001.zip › ijms-1854866-supplementary.pdf]

Supplementary Material

A Novel Pathway of Chlorimuron-ethyl Biodegradation by *Chenggangzhangella methanolivorans* Strain CHL1 and Its Molecular Mechanisms

Materials and Methods

Chemicals

Chlorimuron-ethyl (purity 99.70 %) and reagents for spectroscopic and chromatographic analysis were all purchased from Sigma-Aldrich Chemical Co (Shanghai, China). The spectroscopic and chromatographic analysis reagents were purchased from Sigma-Aldrich Chemical Co (Shanghai, China). Other solvents and chemicals were used as analytical grade, and the enzymes were supplied by TaKaRa Biotechnology (Dalian, China).

Mediums

The components of mineral salts medium (MSM) I were as follows: 2 $g{\cdot}L^{-1}$ $NaNO_3$, 2 $g{\cdot}L^{-1}$ KH_2PO_4, 0.125 $g{\cdot}L^{-1}$ $MgSO_4{\cdot}7H_2O$, 0.5 $g{\cdot}L^{-1}$ NaCl, 0.02 $g{\cdot}L^{-1}$ $FeSO_4{\cdot}7H_2O$, 20 $mL{\cdot}L^{-1}$ methanol, pH 7.0 [31]. The components of MSM II were as follows: 2 $g{\cdot}L^{-1}$ $NaNO_3$, 2 $g{\cdot}L^{-1}$ KH_2PO_4, 0.5 $g{\cdot}L^{-1}$ NaCl, 0.2 $g{\cdot}L^{-1}$ yeast extract, 0.125 $g{\cdot}L^{-1}$ $MgSO_4{\cdot}7H_2O$, 0.02 $g{\cdot}L^{-1}$ $FeSO_4{\cdot}7H_2O$, 20 $mL{\cdot}L^{-1}$ methanol, pH 7.0 [22]. The components of MSM III were as follows: 0.5 $g{\cdot}L^{-1}$ $NaNO_3$, 1 $g{\cdot}L^{-1}$ $(NH4)_2SO_4$, 2.5 $g{\cdot}L^{-1}$ Na_2HPO_4, 1 $g{\cdot}L^{-1}$ KH_2PO_4, 1 $mL{\cdot}L^{-1}$ microelement solution, 1 $mL{\cdot}L^{-1}$ vitamin solution, 1 $mL{\cdot}L^{-1}$ calcium magnesium solution, 20

$mL \cdot L^{-1}$ methanol, pH 7.0. The components of Vitamin solution were as follows: 200 $mg \cdot L^{-1}$ p-aminobenzoic acid, 200 $mg \cdot L^{-1}$ biotin, 200 $mg \cdot L^{-1}$ folic acid, 200 $mg \cdot L^{-1}$ niacin, 100 $mg \cdot L^{-1}$ calcium pantothenate, 100 $mg \cdot L^{-1}$ pyridoxine hydrochloride, 100 $mg \cdot L^{-1}$ riboflavin, 100 $mg \cdot L^{-1}$ thiamine, 1 $mg \cdot L^{-1}$ vitamin B_{12}, filter with 0.22 μm filter membrane, and store at room temperature. The components of calcium and magnesium solution were as follows: 30 $g \cdot L^{-1}$ $CaCl_2$, 20 $g \cdot L^{-1}$ $MgCl_2$. Sterilize at 121 °C for 20 minutes and store at room temperature [6].

Extraction

The collected samples were centrifuged (8000 rpm, 10 min) to remove the bacterial precipitations. Supernatants were collected and extracted by dichloromethane in the same volume for 3 times. The organic phase was combined and dried with N_2 before dissolved with 10ml of methanol, acetonitrile, and deionized water, respectively. The samples were extracted and filtered through 0.22 μm nylon filters.

Transcriptome sequencing

The concentration and purity of RNA were detected by Nanodrop2000 spectrophotometer and RNA integrity was determined with agarose gel electrophoresis [46]. Ribo-Zero Magnetic Kit (Epicentre) was used to remove rRNA, and mRNAs were randomly divided into small fragments by adding fragmentation buffer. Double-stranded cDNA was synthesized by a SuperScript double-stranded cDNA synthesis kit (Invitrogen, CA) with

random hexamer primers (Illumina), and synthesized cDNA was end-repaired, phosphorylated, ‘A’ base added and digested with UNG enzyme before PCR amplification. Range Ultra Agarose was analyzed the products of PCR amplification by Phusion DNA polymerase (NEB) for 15 cycles. After quantified using TBS380, paired-end RNA-seq sequencing library was sequenced by Illumina HiSeq × 10 with read length of 2 × 150 bp [47,48].

Total RNA was extracted from the bacterial precipitations using TRIzol® Reagent (Invitrogen) and genomic DNA was dissolved by DNase I (TaKaRa) according to the manufacturer's instruction. The raw image data was converted into sequence data using base calling. After quality filtration, the remaining rRNA reads were removed by SeqPrep software and modified by adaptor trimming to get clean data.

Real-time quantitative reverse transcription PCR (RT-PCR)

The conditions were as follows: denaturation at 95 °C for 30 s, followed by 40 cycles of 5 s at 95 °C, 5 s at the T_m of the primer pairs used, and 30 s at 60 °C [18].

Gene knockout

Strain CHL1 was cultured in MSM I medium, and the bacterial solution were centrifuged (5000 rpm, 10 min), and washed with 10% glycerin for 3 times to make competent cells. Plasmid pKD46 was transformed into them by Gene Pulser Xcell (Bio-Rad) (25 μF, 1800 V, 200 Ω, 5 ms) to get strain

CHL1[pKD46]. The expression of recombinases was induced with 100 mM L-Arabinose. Kanamycin resistance gene cassette was obtained using PrimeSTAR HS DNA polymerase amplification with primers with homology arms and plasmid pKD4 as templates. And kanamycin resistance gene cassette was transformed into CHL1[pKD46] cells by the same electric shock method as above to obtain the mutants [18]. Homologous recombination was used to replace target gene with kanamycin resistance gene cassette [49]. Plasmid pCP20 was transformed into the mutants to remove kanamycin gene, and then the temperature was raised to 42 °C to remove the plasmid pCP20 to obtain mutants without resistance gene.

Gene complementation

AtzF, *atzD* and *cysJ* amplified by PCR were digested by Xba I and Hind III and then ligated into Xba I and Hind III-digested pEarleyGate100, respectively. Plasmid pEG-*atzF*, pEG-*atzD*, and pEG-*cysJ* was transformed into strains CHL1Δ*atzF*, CHL1Δ*atzD* and CHL1Δ*cysJ* to obtain strains CHL1Δ*atzF*[pEG-*atzF*], CHL1Δ*atzD*[pEG-*atzD*] and CHL1Δ*cysJ*[pEG-*cysJ*], respectively.

Figures

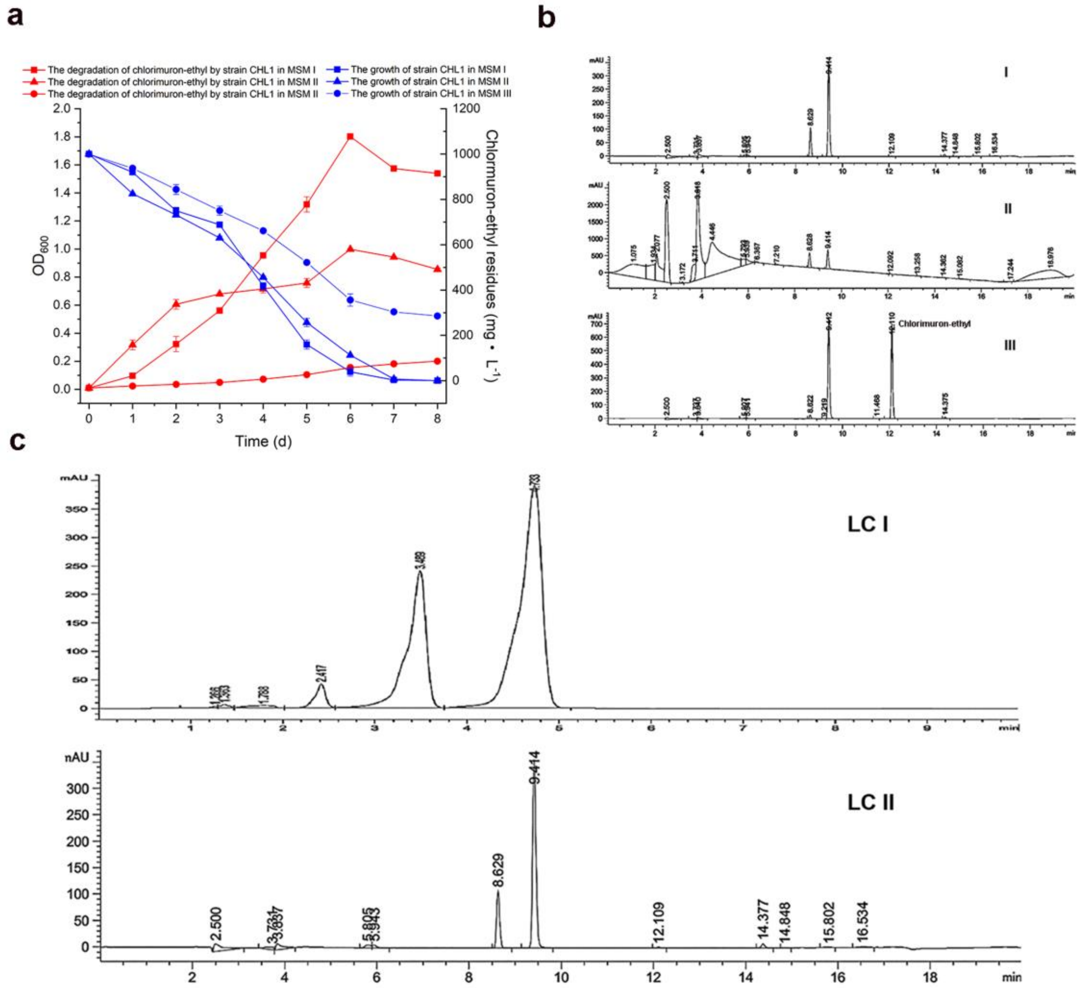

Figure S1. Metabolism of chlorimuron-ethyl by strain CHL1. (**a**) Growth curve and degrading curve of strain CHL1 in mineral salts medium (MSM) I, II III. (**b**) Liquid chromatograms of strain CHL1 culture supernatant in MSM I, II and III on the eighthth day. (**c**) Intermediate degradation products of chlorimuron-ethyl under liquid chromatography (LC) I, II.

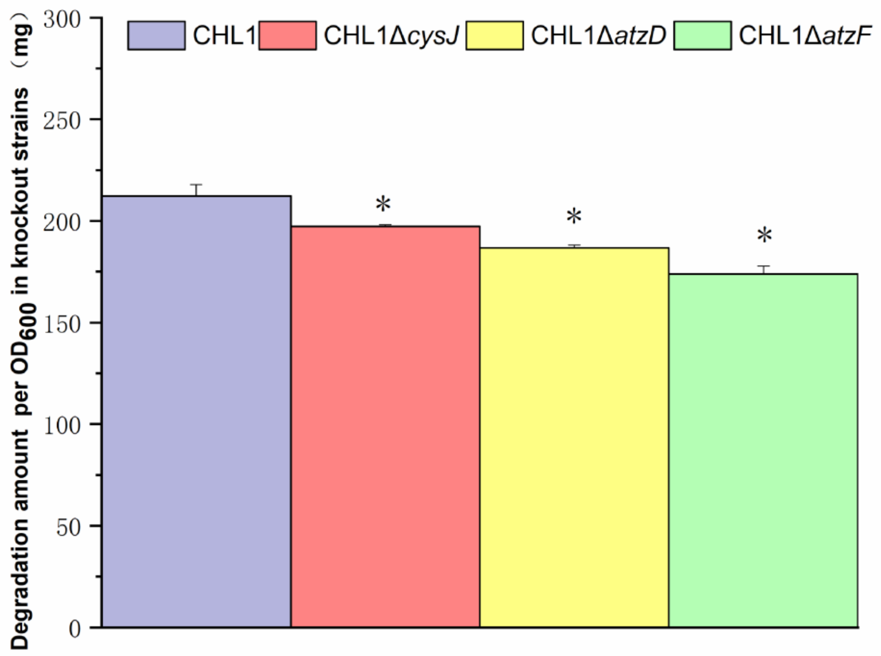

Figure S2. Normalization of the degradation rate of knockout strains. Statistically significant differences between mutant and wild strains are marked * ($p < 0.05$). All assays were performed three replicates.

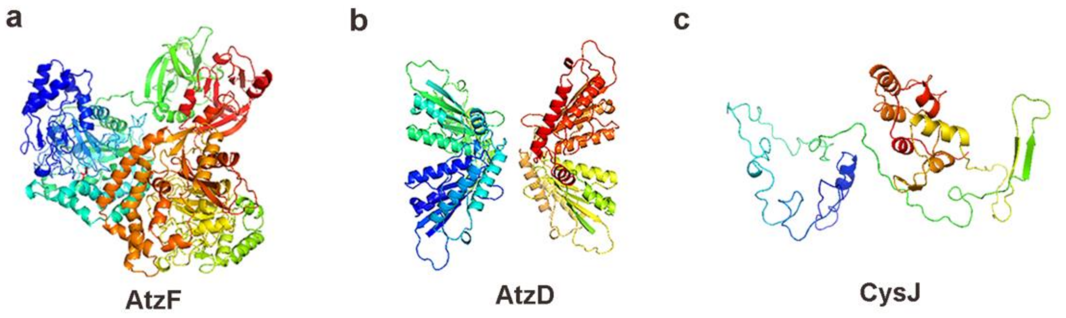

Figure S3. Tertiary structure analysis of AtzF (**a**), AtzD (**b**)and CysJ (**c**) predicted by SWISS-MODEL. The reference tertiary structure model is 6cwj.1 of AtzF (**a**), 3a1i.1 of AtzD (**b**), 6j7i.1 of CysJ (**c**), respectively.

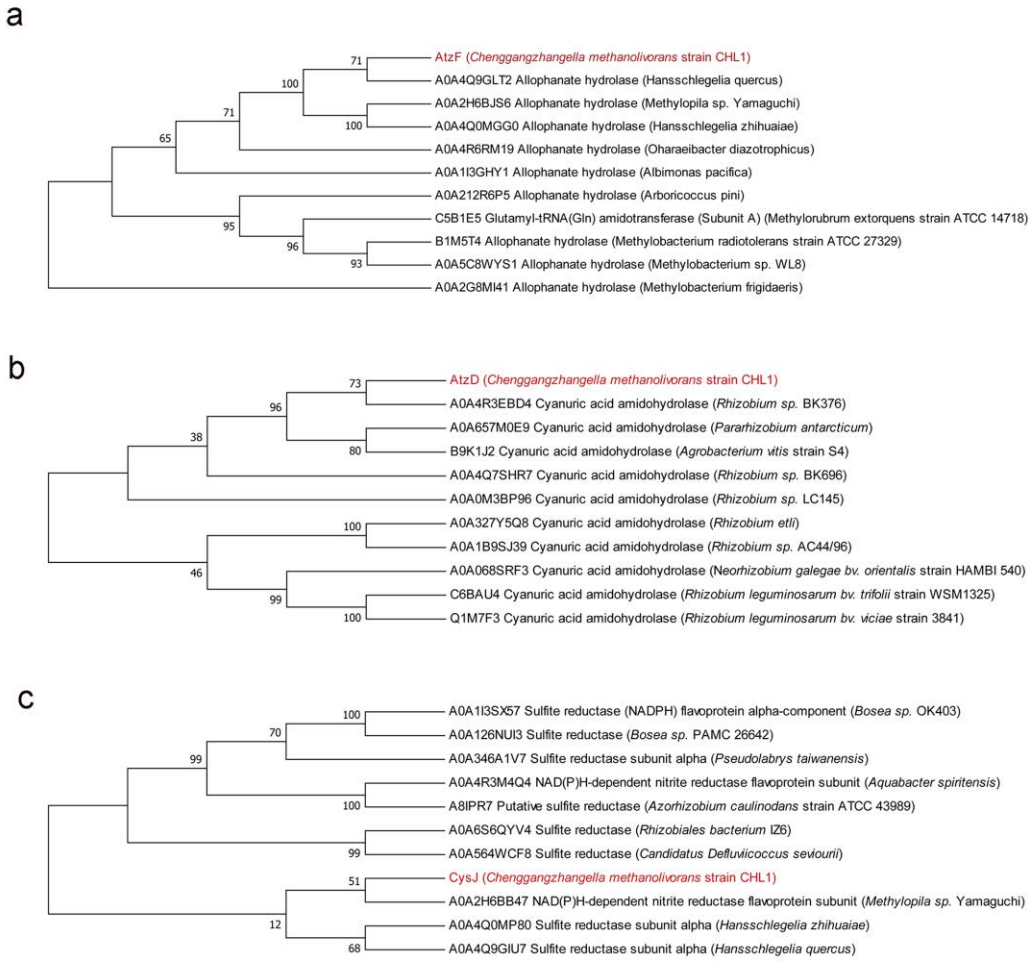

Figure S4. Neighbor-joining phylogenetic trees for AtzF (**a**), AtzD (**b**), and CysJ (**c**). The neighbor-joining phylogenetic trees were constructed using the amino acid sequences. The bootstrap values (expressed as percentages of 1000 replications) are shown at the branch points.

Tables

Table S1. Intermediate degradation products of chlorimuron-ethyl by strain CHL1.

Product	Product mass/ charge	Fragment ion	Fragment ion charge ratio	Fragment ion elemental composition	Product element composition	International Union of Pure and Applied Chemistry (IUPAC) Name
I	274.16	1a	106.21	CHO_3NS^+	$C_{10}H_{11}O_6NS$	[2-(ethoxycarbonyl) benzenesulfonyl] carbamic acid
		1b	230.47	$C_9H_{12}O_4SN^+$		
		1c	255.88	$C_{10}H_{10}O_5NH^+$		
II	159.97	2a	124.06	$C_5H_6ON_3^+$	$C_5H_6ON_3Cl$	4-chloro-6-methoxypyrimidin-2-amine
		2b	128.19	$C_4H_4N_3Cl^+$		
		2c	142.89	$C_5H_4ON_2Cl^+$		
III	386.19	3a	160.18	$C_5H_7ON_3Cl^+$	$C_{13}H_{11}O_6N_4SCl$	2-[[(4-chloro-6-methoxy-2–pyrimidinyl) carbamoyl] sulfamoyl] benzoic acid
		3b	185.73	$C_7H_5O_4S^+$		
		3c	202.73	$C_7H_8O_4NS^+$		
IV	202.06	4a	122.34	$C_7H_7O_2^+$	$C_7H_7O_4NS$	2-sulfamoyl benzoic acid
		4b	184.92	$C_7H_5O_4NS^+$		
V	185.81	5a	107.96	$C_7H_6O^+$	$C_7H_6O_3NS$	3-hydroxy-2,3-dihydro-1λ^6,2-benzothiazole-1,1-dione
		5b	169.88	$C_7H_6O_3S^+$		
VI	340. 15	7a	228.15	$C_8H_6O_5NH^+$	$C_{12}H_{12}O_6N_4S$	2-{[({[(1-hydroxy-3-iminoprop-1-en-1-yl) imino] methyl} carbamoyl) amino] sulfinyl} benzoic acid
		7b	322.16	$C_{12}H_{12}O_5N_4S^+$		
VII	302.07	8a	228.75	$C_8H_6O_5NH^+$	$C_{10}H_{11}O_6N_3S$	2-{[({[(1-hydroxy-3-iminoprop-1-en-1-yl) imino] methyl} carbamoyl) amino] sulfinyl} benzoic acid
		8b	241.05	$C_8H_8O_5NS^+$		
		8c	284.31	$C_{10}H_{11}O_5N_3S^+$		
VIII	284.19	9a	122.96	$C_7H_6O_2^+$	$C_{10}H_{11}O_5N_3S$	2-[({[(methylideneamino) methyl] carbamoyl} amino) sulfinyl] benzoic acid
		9b	228.87	$C_8H_6O_5NH^+$		

Table S2. RNA-seq basis analysis of strain CHL1

Sample Name	Raw reads (bp)	Clean Reads (bp)	Clean Q20 (%)	Clean Q30 (%)
A control	16,367,725±1,026,657	14,940,667±714,710	99.20±0.04	97.31±0.12
A treatment	14,931,122±1,089,425	14,118,074±466,335	99.21±0.01	97.35±0.02
B control	22,914,114±1,013,679	22,600,503±993,913	99.25±0.01	97.46±0.02
B treatment	21,953,072±724,590	21,687,283±734,703	99.24±0.01	97.42±0.04
C control	22,936,105±1,182,851	22,664,187±1,169,888	99.31±0.01	97.63±0.04
C treatment	22,114,054±2,642,726	21,844,017±2,640,375	99.28±0.05	97.56±0.15

Clean Q_{20} (%) and Clean Q_{30} (%) indicate that the Phred value after data quality control was greater than 20 and 30 bases as a percentage of the total bases, respectively. The values in the table were the average values of the sample A, sample B and sample C.

Table S3. Genome mapped reads and CDS mapped reads

Sample Name	Genome Mapped Reads (bp)	Genome Mapped Ratio (%)	CDS Mapped Reads (bp)	CDS Mapped Ratio (%)
A control	13,900,900±1,368,135	92.81±4.84	11,943,170±1,161,165	79.74±4.06
A treatment	13,176,837±143,370	93.43±3.19	11,351,855±110,826	80.49±2.64
B control	22,366,301±995,153	98.96±0.05	19,060,975±822,903	84.34±0.26
B treatment	21,340,137±817,905	98.38±0.69	18,239,278±806,240	84.08±1.02
C control	22,397,175±1,150,639	98.82±0.09	18,063,111±955,593	79.69±0.12
C treatment	20,941,718±3,256,703	95.48±4.26	17,370,226±2,742,782	79.17±3.64

Table S4. Basic information on putative chlorimuron-ethyl degradation genes in strain CHL1

Enzyme name	Annotation	Locus_tag (NCBI)	Log_2FC			KEGG
			Sample A	Sample B	Sample C	
GST	Glutathione S-transferase	K6K41_17220	-0.18	-0.49	0.88*	Drug metabolism - cytochrome P450<br>Glutathione metabolism
CopA	Dehalogenase	K6K41_23445	0.14	3.79*	-0.10	MAPK signaling pathway – plant<br>Platinum drug resistance
CrtD	Desaturase	K6K41_01655	-0.53	0.46*	-0.08	Carotenoid biosynthesis
CrtC	Hydratase	K6K41_01650	1.50*	0.01	0.48	Carotenoid biosynthesis
Limb	Monooxygenase	K6K41_27575	0.07	0.23	0.99*	Limonene and pinene degradation

Statistically significant differences were marked with * (False discovery rate FDR < 0.05). If the value of Log_2FC was greater than 0, it indicated up-regulated. If the value of Log_2FC was less than 0, it indicated down-regulated.

Table S5. The plasmids and bacterial strains used in this study

Strains and plasmids		Relevant genotype or characteristic
Plasmid	pKD4	Knockout vector, Kan^R
	pKD46	Knockout vector, Amp^R
	pCP20	Knockout vector, Amp^R
	pEarleyGate100	Complementation vector, Kan^R
	pET-28a (+)	Expression vector, Kan^R
	pEG-*atzF*	*AtzF*, *atzD* and *cysJ* complementation
	pEG-*atzD*	vectors by pEarleyGate100,
	pEG-*cysJ*	respectively.
Chenggangzhangella	CHL1	Chlorimuron-ethyl utilizer, wild type
methanolivorans	CHL1Δ*atzF*	
	CHL1Δ*atzD*	Strain CHL1 with *atzF*, *atzD, cysJ* and,
	CHL1Δ*cysJ*	*carE* deleted, respectively.
	CHL1Δ*carE*	
	CHL1Δ*atzF*[pEG-*atzF*]	*AtzF*, *atzD* and *cysJ* was complemented
	CHL1Δ*atzD*[pEG-*atzD*]	by pEG-atzF, pEG-*atzD* and pEG-*cysJ*
	CHL1Δ*cysJ*[pEG-*cysJ*]	plasmids in CHL1Δ*atzF*, CHL1Δ*atzD*
		and CHL1Δ*cysJ* strains, respectively.
	CHL1[pKD46]	Strain CHL1 with plasmid pKD46

Table S6. The primer sequences used in this study

Primers	Sequences (5′-3′)
FKF-*atzF*-F	CCCCTCGCCCATCCGCTCGCGCGCCTCGCGAACCGAGGCCACGTCGATCGCGTCGCCCATGTGTAGGCTGGAGCTG
FKF-*atzF*-R	GCCGAGCAGCGCGGCGGCGGCGTCGAGATGCGCTTCGAGCGCGGCGGCGTCGTCGGCCAATCCTCCTTAGTTCCTATTCC
FKF-*atzD*-F	GCAGGGCGGCGTGTTCGGGACGGTCGCGACTTCGGACGCCTTCATCCGGGCGATCGCCTGTGTGTAGGCTGGAGCTG
FKF-*atzD*-R	CCCCTCGCCCATCCGCTCGCGCGCCTCGCGAACCGAGGCCACGTCGATCGCGTCGCCCAATCCTCCTTAGTTCCTATTCC
FKF-*cysJ*-F	GGAGCGTCGCAGGCGCGTCTCGAAGCCAGCAGTGTCCTCTCCGATCGTCGGAGCACGTCCTGTGTAGGCTGGAGCTG
FKF-*cysJ*-R	TAGAAGAAGTCGGTCGCCTCGCGCTGGTGGCCGAAGAACAGCCACGCGCCGCCGTCGCTATCCTCCTTAGTTCCTATTCC
atzF(XH)-F	GCTCTAGAGATCGTCTCCAACCCCTGCTTC
atzF(XH)-R	CCCAAGCTTCGGGCTCCATCTCGTCCTC
atzD(XH)-F	GCTCTAGAATGGCGTCGATGCGCGCC
atzD(XH)-R	CCCAAGCTTTCACCGCATGGTCGATCGCGAG
cysJ(XH)-F	GCTCTAGAATGACCGCCCAGACCCCGAT
cysJ(XH)-R	CCCAAGCTTCATGCGGTCCTGGACGTAGA
Q-*atzD*-F	GGCTGCGTCAACGACTTCACC
Q-*atzD*-R	TCCGTACCGCCCGACATCAC
Q-*atzF*-F	CCAACGCCGACGACATCCATC
Q-*atzF*-R	GAGTTCATACATGCCGCCGAAGG
Q-*cysJ*-F	GATCTGATGAGCCTCGACGTTCTG
Q-*cysJ*-R	GCGCGGACGAGATCGAATATAGC
Q-16S rRNA-F	CCTACGGGAGGCAGCAG
Q-16S rRNA-R	ATTACCGCGGCTGCTGG

“FKF-X” represents the primers of gene knockout. “X(XH)” represent the primers of gene complementation, and “XH” represents the restriction sites of Xba I and Hind III. “Q-X” represents the primers of quantitative RT-PCR.
